# How does active yeast supplementation reduce the deleterious effects of aflatoxins in Wistar rats? A radiolabeled assay and histopathological study

**DOI:** 10.1007/s11274-024-03981-5

**Published:** 2024-04-17

**Authors:** Pietro Sica, Maria Antonia Domingues, Layna Amorim Mota, Alana Uchôa Pinto, Ana Angelita Sampaio Baptista, Jorge Horii, Adibe Luiz Abdalla, Antonio Sampaio Baptista

**Affiliations:** 1 Department of Plant and Environmental Sciences, University of Copenhagen, 40 Thorvaldsenvej, Frederiksberg, 1870 Denmark; 2 Department of Agri-food Industry, Food and Nutrition, College of Agriculture “Luiz de Queiroz”, University of Sao Paulo”, Padua Dias Avenue, Piracicaba, Sao Paulo, 13418-900 Brazil; 3 Department of Preventive Veterinary Medicine, State University of Londrina, Londrina, Parana 86057-970 Brazil; 4 Center for Nuclear Energy in Agriculture (CENA), University of Sao Paulo, 303, Centenario Avenue, Piracicaba, Sao Paulo, 13400-970 Brazil

**Keywords:** Probiotics, *Saccharomyces cerevisiae*, Aflatoxicosis, Aflatoxin metabolism, Radiolabeled, Mycotoxins

## Abstract

The aim of this study was to investigate the mechanisms by which yeasts (*Saccharomyces cerevisiae)* control the toxic effects of aflatoxins, which are not yet fully understood. Radiolabeled aflatoxin B_1_ (AFB_1_^3^H) was administered by gavage to Wistar rats fed with aflatoxin (AflDiet) and aflatoxin supplemented with active dehydrated yeast Y904 (AflDiet + Yeast). The distribution of AFB_1_^3^H and its metabolites were analyzed at 24, 48 and 72 h by tracking back of the radioactivity. No significant differences were observed between the AflDiet and AflDiet + Yeast groups in terms of the distribution of labeled aflatoxin. At 72 h, for the AflDiet group the radiolabeled aflatoxin was distributed as following: feces (79.5%), carcass (10.5%), urine (1.7%), and intestine (7.4%); in the AflDiet + Yeast the following distribution was observed: feces (76%), carcass (15%), urine (2.9%), and intestine (4.9%). These values were below 1% in other organs. These findings indicate that even after 72 h considerable amounts of aflatoxins remains in the intestines, which may play a significant role in the distribution and metabolism of aflatoxins and its metabolites over time. The presence of yeast may not significantly affect this process. Furthermore, histopathological examination of hepatic tissues showed that the presence of active yeast reduced the severity of liver damage caused by aflatoxins, indicating that yeasts control aflatoxin damage through biochemical mechanisms. These findings contribute to a better understanding of the mechanisms underlying the protective effects of yeasts against aflatoxin toxicity.

**Graphical abstract:**

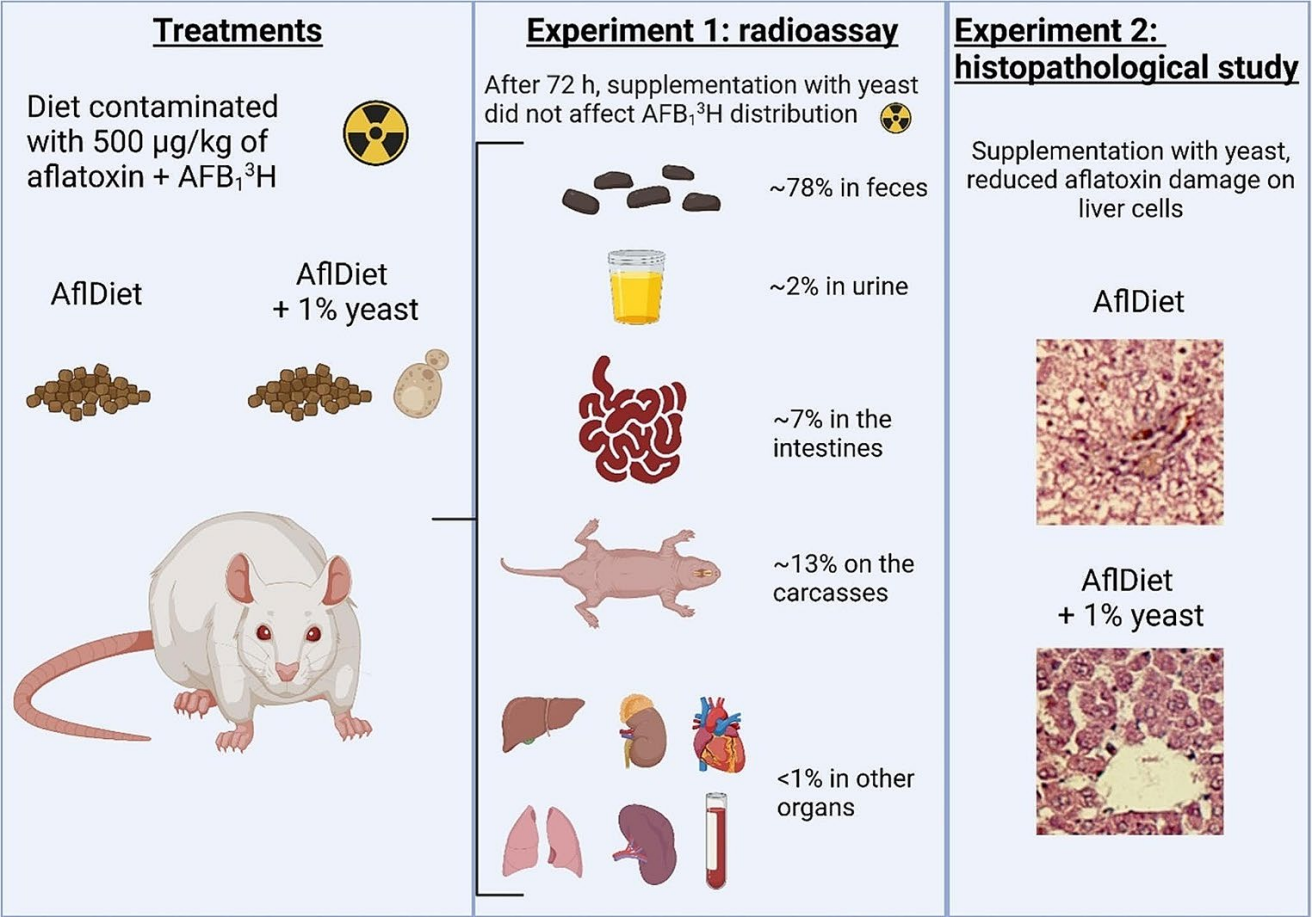

## Introduction

The yeast *Saccharomyces cerevisiae* is a unicellular microorganism that is widely used in the production of various products, such as bread, wine, beer (Johnson and Echavarri-Erasun 2011), biofuels (Sica et al. 2021), and others. In many of these processes, yeast acts as a transformative agent and can be manipulated and repurposed after use, resulting in an abundant supply of this important biological agent worldwide (Johnson and Echavarri-Erasun 2011). In addition to that, *Saccharomyces cerevisiae* is considered the best-studied eukaryote, with well-known genetic (Goffeau et al. 1996), physiological, and biochemical characteristics, making yeast one of the primary species of interest for biotechnological applications (Johnson and Echavarri-Erasun 2011).

Yeast has various applications, including as a probiotic food supplement through the inoculation of active cells (Staniszewski and Kordowska-Wiater 2021). The aim of this is to positively impact the host and promote a healthy balance in the intestinal microbiota. Probiotics offer several benefits to the individual, including increased resistance to infectious diseases (Rostami et al. 2018), reduced risk of diarrhea (Liu et al. 2022), and lowered blood pressure (Dong et al. 2013). Additionally, probiotics stimulate phagocytosis by peripheral leukocytes in the blood (Schiffrin et al. 1995) and anticarcinogenic effects (Kato et al. 1981; Kumar et al. 2012). In animal production, yeast supplementation has also been shown to increase animal live weight (Bontempo et al. 2006), improve food digestion efficiency (Fuller 1992), boost production (Alagawany et al. 2023), and can reduce toxic effects caused by aflatoxins (Baptista et al. 2008).

The ingestion of food contaminated with aflatoxins is a growing concern worldwide in terms of animal and human health (Reddy et al. 2010; Kumar et al. 2017) due to its potential carcinogenic, mutagenic (IARC 2002; Liu and Wu 2010), teratogenic (IARC 2002; Silva et al. 2021), and lethal effects even at lower doses (Mohd-Redzwan et al. 2013). To mitigate the damage caused by these toxins, various strategies have been developed over the past decades, including biological control by supplementing yeast (Afzal et al. 2022). This technique has proven to be one of the most effective ways of reducing the deleterious effects of aflatoxins, as demonstrated by previous studies (Stanley et al. 1993; Çelik et al. 2001; Galvano et al. 2001; Parlat et al. 2001; Baptista et al. 2004; Shetty and Jespersen 2006; Shetty et al. 2007).

Furthermore, studies have demonstrated that active dehydrated *Saccharomyces cerevisiae*, when inoculated at doses ranging from 0.01 to 1%, can control aflatoxicosis - a condition caused by the ingestion of aflatoxins. By inoculating active yeast into the diets of individuals that received aflatoxin-contaminated food, there were significant reductions in the harmful effects caused by the ingestion of these toxins, as demonstrated by histopathological studies and biochemical trials (Stanley et al. 1993; Parlat et al. 2001; Baptista et al. 2002; Shetty and Jespersen 2006).

Previous studies have only shed light on the potential of *Saccharomyces cerevisiae* in controlling hepatic damage caused by aflatoxins. However, the mechanism behind this ability remains unclear and several hypotheses have been proposed. In order to contribute to the understanding of this mechanism, in the present study we carried out two bioassays with Wistar rats with the following objectives:

1. Experiment 1 – radioassay: to evaluate the absorption, distribution, and excretion of radioactivity from AFB_1_^3^H;
2. Experiment 2 – histopathological study: to investigate the effect of yeast Y904 supplementation on reducing hepatic damages due to aflatoxicosis.

Through these experiments, we expected to gain new insights into the potential mechanisms underlying the ability of yeast to prevent or mitigate the harmful effects of aflatoxins.

## Materials and methods

### Reagents

The standard labeled aflatoxin (AFB1^3^H) was purchased from Moravek Biochemical (Brea, California, USA). Other standards of aflatoxins B_1_, B_2_, G_1_ and G_2_ were acquired from Sigma Aldrich (Sao Paulo, Brazil). Insta Gel solution was purchased from Pelkin Elmer Radiochemicals (Boston, MA, USA). Individual doses were prepared from a standard solution of AFB1^3^H (9.25 × 10^6^ Bq) labeled aflatoxin B1, containing 4.7 µg of aflatoxin B_1_ (16.6 Ci/mmol) diluted in 0.5 mL of 8% sulphoxide dimethyl (Moravek Biochemical, Brea, California, USA). Hereafter, AFB1^3^H refers to Aflatoxin B1 radiolabeled with ^3^H. Bq (Becquerel) is the international system of units for radioactivity. One Bq is the activity of a quantity of radioactive material in which one nucleus disintegrates per second.

The other reagents and solutions used in this study were purchased from Merck S.A. (Sao Paulo, Brazil).

### Biological assays

Forty-two male albino Wistar rats (*Rattus norvegicus*) aged 21 to 25 days, weighing approximately 60 g each, were used in these experiments.

Throughout the experimental period, the animals received the same diet based on proteins, carbohydrates, lipids, fibers, mineral mixture, and vitamin mixture (diet AIN-93G), described by (Reeves et al. 1993), and adjusted to each treatment, with the daily supply of “*ad libitum”* water and 15 g of food to each animal. Diet contamination was carried out by adding rice meal that was naturally contaminated with aflatoxins. All diets were stored in a cold chamber (4 ± 1 °C) until offered to the animals.

Active dehydrated yeast (*Saccharomyces cerevisiae*, Y904) was added to the diets after the contamination. The viability of the yeast cells was determined as 98.7% at the time of the experiment, by the blue methylene coloring method (0.1%), as described by Pierce (1970).

The aflatoxin analyzes were performed as previously adapted by Baptista et al. (2008). The total aflatoxin diet contamination was B1 = 470 µg kg^− 1^, B2 = 30 µg kg^− 1^, G1 and G2 = not detected. The limit of detection for each aflatoxin was 0.5 µg kg^− 1^. Zearalenone, ochratoxin A and deoxynivalenol were also evaluated (Baptista et al. 2008) and these substances were not detected. The limit of detection for these mycotoxins was 5 µg kg^− 1^, 60 µg kg^− 1^, and 50 µg kg^− 1^, respectively.

#### Experiment 1 – radioassay

In the first experiment, 18 rats were divided into two groups of nine each and placed into individual cages distributed in an experiment split plot (with the time factor considered as a plot and the treatment factor as subplots). Each group received the following diets: Group A – basal diet contaminated with 500 µg kg^− 1^ of aflatoxins (AflDiet – aflatoxins control); Group B – basal diet contaminated with 500 µg kg^− 1^ of aflatoxins supplemented with 1% of dehydrated active yeast strain Y904 (AflDiet + Yeast) (Table 1).

**Table 1:** Description of treatment used in both experiments carried out in this study: radioassay (1) and histopathological study (2)

	**Experiment 1 - Radioassay**	
Split plot design (time = plot; treatment = subplots).		
AflDiet		500 µg kg^− 1^ of aflatoxin supplemented in the diet
AflDiet + Yeast		500 µg kg^− 1^ of aflatoxin plus 1% yeast supplemented in the diet
		**Experiment 2 - Histopathological study**
Completely randomized design		
NAflDiet		normal diet with aflatoxin at non-detectable levels (≤ 0.5 µg kg^− 1^)
AflDiet		500 µg kg^− 1^ of aflatoxin supplemented in the diet
AflDiet + Yeast		500 µg kg^− 1^ of aflatoxin plus 1% yeast supplemented in the diet

Wistar rats with 21-day-old were transferred to metabolic cages as soon as they arrived at the laboratory, where they remained for 12 days for acclimation period, receiving diets described for groups A and B (1st experiment).

On the 12th day, each animal received a single dose of 12.58 × 10^4^ Bq of AFB1^3^H orally by gavage. The 12-day period was chosen based on previous studies (Baptista et al. 2008) to ensure that the animals were beginning to show the adverse effects of the aflatoxin in their diet. The radioisotopes were administered with a single dose of radioisotopes by gavage to ensure that all the radiolabeled aflatoxin was ingested and to facilitate the tracking of the radiomolecules in the animal’s body at different sampling times.

At each time of data collection (24, 48 and 72 h after application of radiomolecule), three animals of each treatment were sacrificed. Samples of urine, feces, heart, liver, spleen, lungs, kidneys, gastrointestinal tract, blood, and carcasses of each animal were collected. Excretions (feces and urine) were removed at each sampling period to ensure that the amount of material obtained represented only the interval between sample collections. The samples were frozen at -20 ± 1 °C until analysis.

The distribution of the radiomolecules in the animals was analyzed by the detection of radioactivity found in several collected materials, according to (Helferich et al. 1986). The samples (50 to 300 mg) were subjected to combustion in an oxidizer model “Biological Oxidizer Ox 500”, of R.J. Harvey Instruments Corporation. The tritium from each sample was collected in 15 ml of water. After that, 10 ml of sample was transferred to scintillation vials containing 10 ml of scintillation solution (Insta Gel Plus). The specific activity was determined by liquid scintillation, using a scintillation spectrometer model “Packard Tri-Carb 1600 TR”. The counting time for each sample was 5 min.

#### Experiment 2 – histopathological study

In the second experiment, in a delineation entirely randomized, 24 animals were divided into three groups of eight individuals each; which were distributed in individual cages and during 28 days were submitted to the following treatments: NAflDiet – aflatoxin-free basal diet (at non-detectable levels (ND); AflDiet – basal diet contaminated with 500 µg kg^-1^ of aflatoxins; and AflDiet + Yeast – basal diet contaminated with 500 µg kg^-1^ of aflatoxins supplemented with 1% of yeast of the strain Y904, active dehydrated (Table 1).

At 28 days after the beginning of the experiment, eight animals of each treatment were anesthetized in a chamber saturated with halothane. Their abdominal cavities were opened and their internal organs were removed. Liver fragments were fixed in 10% formaldehyde and used for the histopathological examination.

From each piece of hepatic tissue of the 24 animals, three histological blades were prepared according to the procedures described by (Pacheco 1981) and stained with hematoxylin and eosin (HE). After the preparation, the blades were submitted for the histopathologic study under light microscopy at a magnification of 16 × 1.25 × 10 (200 x). Hepatic tissues that received the aflatoxin-free diet were used as reference standards for hepatotoxicity.

### Statistical analysis

The experimental delineation used in the first experiment was a split plot design, with the time factor as plot and the treatment factor as subplots. The results obtained in both experiments were analyzed using SAS (Herzberg 1990) for determining the variance analysis. For the second experiment, experimental delineation was applied entirely randomly, and the analyses were qualitative.

## Results

### Radioassay - Experiment I

#### Distribution of radiolabeled AFB_1_^3^H in rat carcasses over time

**Fig. 1:**
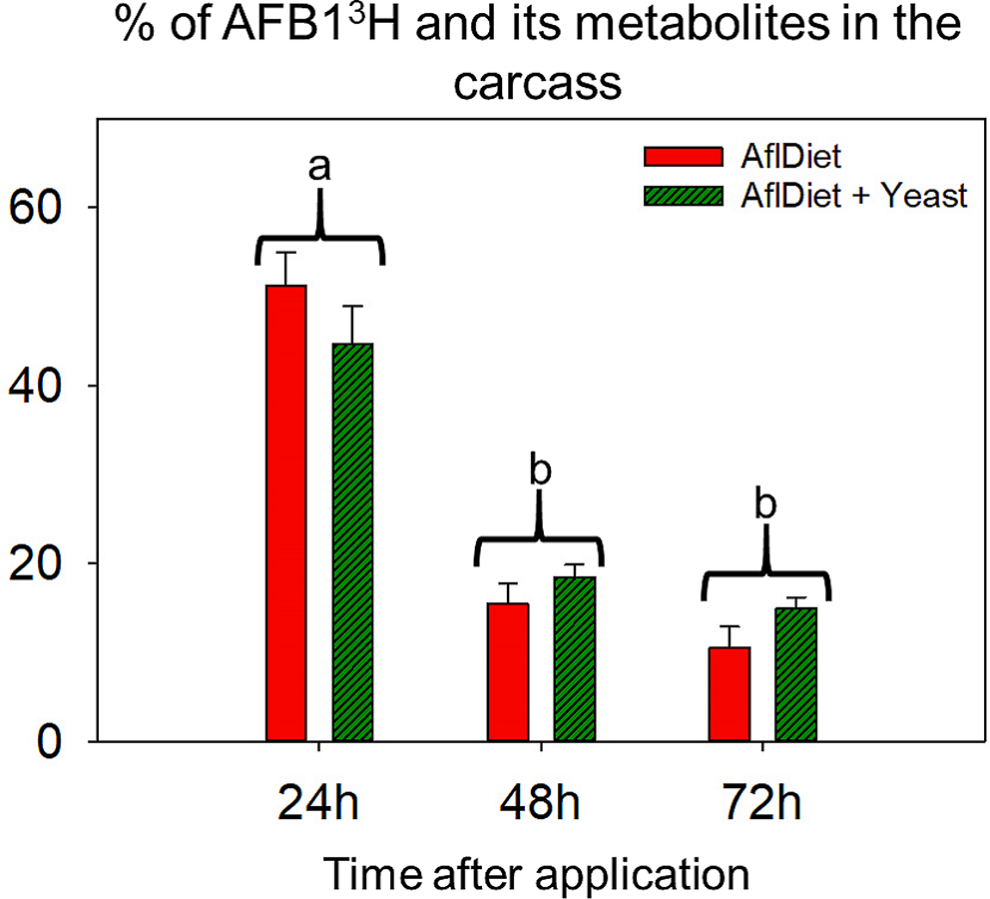
Percentage (%) of total applied radiolabeled AFB1^3^H and/or their metabolites on Wistar rats’ carcasses at 24 h, 48 h, and 72 h after application (*n* = 3). The treatments with aflatoxin (AflDiet, red) and aflatoxin plus 1% yeast (AflDiet + Yeast, dark green) are represented. Error bars represent the standard error. ANOVA test did not indicate significant difference (*p* > 0.05) between treatments at the same time point. Different letters indicate a significant difference between time points (*p* < 0.05)

The radioactivity values in the carcasses at the same sampling time point did not show a significant difference between the two treatment groups (AflDiet vs. AflDiet + Yeast), as determined by ANOVA test (*p* > 0.05). However, the distribution of radiolabeled aflatoxin B1 (AFB1) in the rat carcasses differed over time. At 24 h after the labeled aflatoxin application, 51.2% and 44.7% of the total radiomolecules were detected in the carcasses of rats in the AflDiet and AflDiet + Yeast groups, respectively. This percentage decreased to 15.5% and 18.5% at 48 h, and to 10.5% and 15% at 72 h after the application of radiomolecules (Fig. 1).

#### Distribution of radiolabeled AFB1^3^H in rats’ intestines over time

**Fig. 2:**
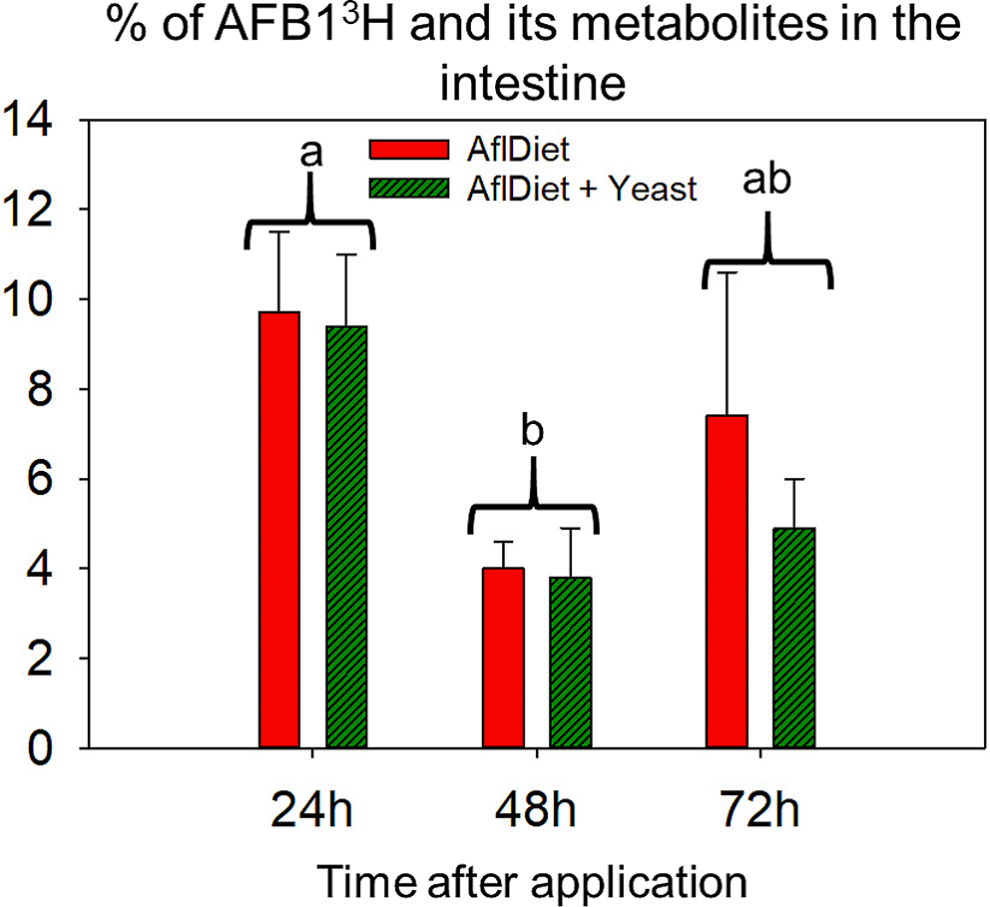
Percentage (%) of total applied radiolabeled AFB1^3^H and/or their metabolites on Wistar rats’ intestines at 24 h, 48 h, and 72 h after application (*n* = 3). The treatments with aflatoxin (AflDiet, red) and aflatoxin plus 1% yeast (AflDiet + Yeast, dark green) are represented. Error bars represent the standard error. ANOVA test did not indicate significant difference (*p* > 0.05) between treatments at the same time point. Different letters indicate a significant difference between time points (*p* < 0.05)

The radioactivity values in the intestines did not differ significantly between the two treatment groups (AflDiet vs. AflDiet + Yeast) at the same time points, as determined by ANOVA test (*p* > 0.05). The highest values of radiolabeled aflatoxin and/or its metabolites in the intestines were observed 24 h after the application, for both treatments, with 9.7% and 9.4% for AflDiet and AflDiet + Yeast, respectively. A significant reduction was observed at 48 h in both treatments, with 4% and 3.8%, respectively (Fig. 2). These results suggest that the intestines may play a significant role in the distribution and metabolism of radiolabeled aflatoxin and its metabolites in the rats over time and that the presence of yeast may not have a significant effect on this process.

#### Excretion of radiolabeled AFB1^3^H in rats’ feces over time

**Fig. 3:**
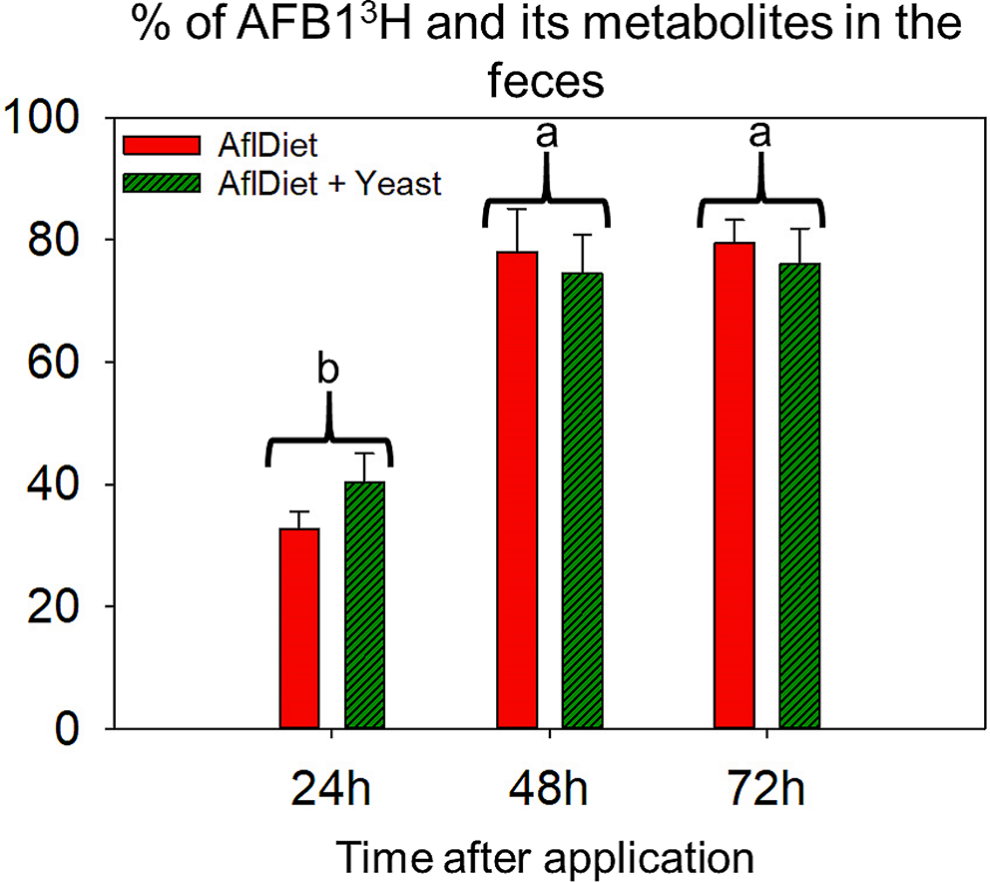
Percentage (%) of total applied radiolabled AFB1^3^H and/or their metabolites on Wistar rats’ feces at 24 h, 48 h, and 72 h after application (*n* = 3). The treatments with aflatoxin (AflDiet, red) and aflatoxin plus 1% yeast (AflDiet + Yeast, dark green) are represented. Error bars represent the standard error. ANOVA test did not indicate significant difference (*p* > 0.05) between treatments at the same time point. Different letters indicate a significant difference between time points (*p* < 0.05)

The radioactivity values excreted in feces did not differ significantly between the two treatment groups (AflDiet vs. AflDiet + Yeast) at the same sampling time points, as determined by ANOVA test (*p* > 0.05). High levels of radioactivity were already detected in feces 24 h after the application of radiolabeled aflatoxin (AFB1^3^H) for both treatments AflDiet and AflDiet + Yeast, with 32.7% and 40.3% of the applied radioactivity being excreted in the feces, respectively. At 48 h, these values increased to 78% and 74.5% for both treatments, respectively, and at 72 h, the values were 79.5% and 76%, respectively (Fig. 3). These results suggest that a significant proportion of the radiolabeled aflatoxin and its metabolites are excreted in feces already at 24 h and most of it at 48 h. The presence of yeast may not have a significant effect on this process.

#### Distribution of radiolabeled AFB1^3^H in rats’ urine and other organs over time

No significant differences were observed in the radioactivity values of rats’ urine and other organs between the AflDiet and AflDiet + Yeast treatment groups at the same sampling time points, as determined by ANOVA test (*p* > 0.05). At 24 h, 2.7% and 2.0% of the total radioactivity was recovered in the urine of rats subjected to AflDiet and AflDiet + Yeast, respectively. At 48 h, these values were 1.4% and 2% in urine samples, respectively, and 1.7% and 2.9% were recovered in urine samples at 72 h, respectively (Table 2). These results suggest that the presence yeast did not have a significant effect on the excretion of radiolabeled aflatoxin and its metabolites through urine.

**Table 2:** Percentage (%) of total applied radiolabeled AFB1^3^H and/or their metabolites on Wistar rats’ urine, kidneys, blood, lungs, spleen, heart, and liver at 24 h, 48 h, and 72 h after application (*n* = 3). The treatments with aflatoxin (AflDiet) and aflatoxin plus 1% yeast (AflDiet + Yeast) are represented. ANOVA test did not indicate significant differences (*p* > 0.05) between treatments at the same time point

		24 h			48 h			72 h	
		AflDiet	AflDiet + Yeast		AflDiet	AflDiet + Yeast		AflDiet	AflDiet + Yeast
	----------------------------------------------------------%---------------------------------------------------								
Urine		2.7	2.0		1.4	2.0		1.7	2.9
Kidneys		0.6	0.5		0.1	0.2		0.1	0.2
Blood		0.7	0.7		0.2	0.3		0.4	0.3
Lungs		0.1	0.1		0.03	0.03		0.02	0.03
Spleen		0.3	0.2		0.1	0.1		0.04	0.1
Heart		0.3	0.4		0.1	0.1		0.1	0.1
Liver		1.8	1.5		0.6	1.0		0.4	0.5

The livers at 24 h showed higher average values (> 1%) compared to other organs (kidneys, lungs, spleen, heart, and blood) analyzed in this study. However, at 48 and 72 h, the radioactivity values for all the organs and blood were below 1% (Table 2). The recovery of radioactivity in other organs was also similar between the two treatment groups, indicating that the yeast may not affect the distribution or accumulation of the radiolabeled aflatoxin in different tissues.

### Histopathologic study – experiment 2

The bioassay with the histopathological examination of hepatic tissues of the animals under different treatments (Table 1) was a complementary study to the radioassay, with the objective to assess the effects of yeast on damages caused to the cell by aflatoxins.

**Fig. 4:**
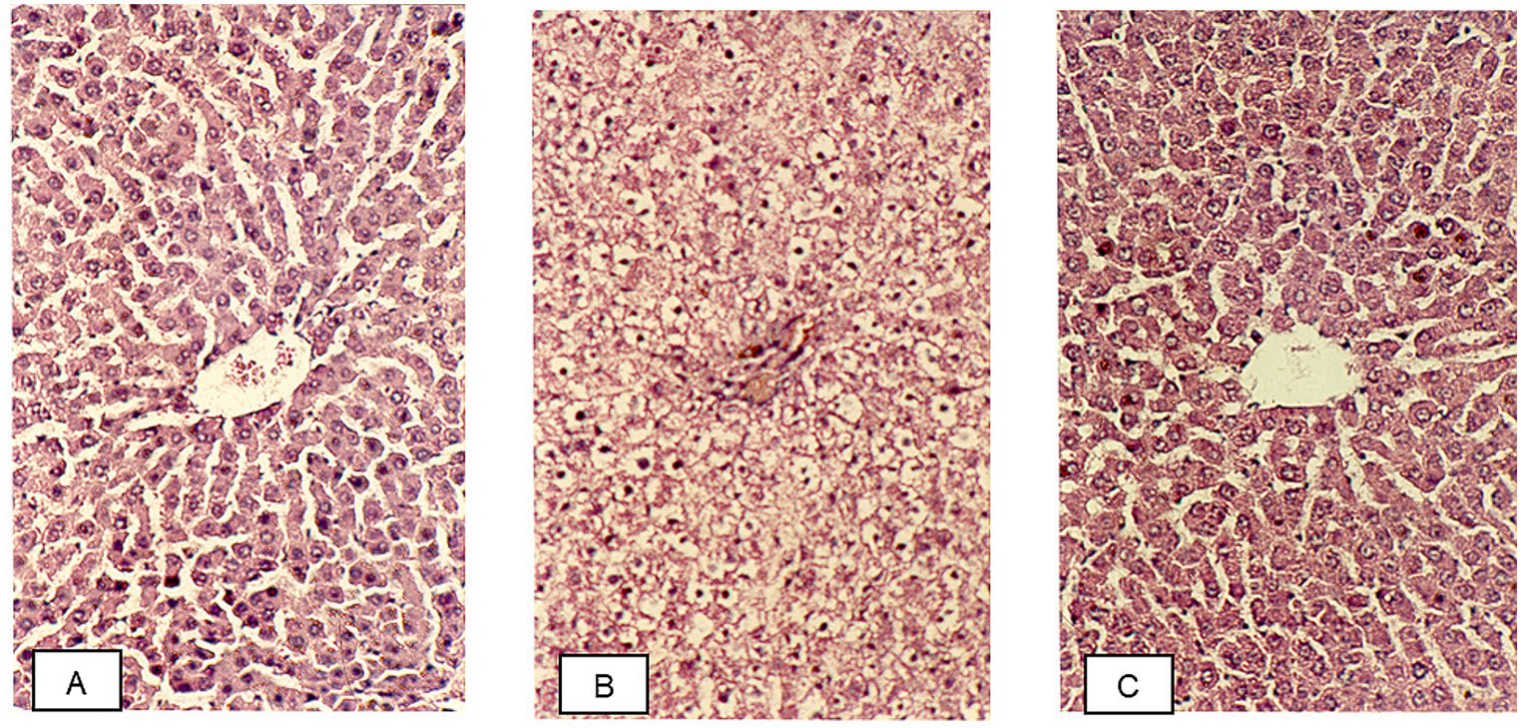
Microphotography (200x) of the hepatic tissue of: (**A**) a rat that received aflatoxin-free diet (NAflDiet); (**B**) a rat that received a diet contaminated with 500 µg kg^− 1^ of aflatoxins (AflDiet); (**C**) a rat that received a diet contaminated with with 500 µg kg^− 1^ of aflatoxins and supplemented with 1% of active yeast (AflDiet + Yeast)

Liver tissues from rats fed yeast-free diets showed normal cell organization with well-organized sinusoidal capillaries and a few cells undergoing physiological degeneration due to aging. As expected, these rats did not exhibit signs of hepatotoxicity since they were not exposed to aflatoxins. These tissues were used as a reference standard for the other treatment groups (Fig. 4A). In contrast, rats fed diets contaminated with 500 µg kg^− 1^ of aflatoxins (AflDiet) showed severe hepatotoxic effects with necrosis in most cells, hydropic degeneration, and vacuolization, indicating intense damage to the liver tissue (Fig. 4B). However, rats fed diets contaminated with 500 µg kg^− 1^ of aflatoxins supplemented with 1% active yeast (AflDiet + Yeast) showed a slight cell disorganization and no necrotic cells. The sinusoidal capillaries were well-organized, and the cells appeared integral without signs of degeneration, except for normal physiological occurrences (Fig. 4C). This suggests that yeast supplementation may have attenuated the hepatotoxic effects of aflatoxins.

## Discussion

### Aflatoxin excretion on urine and feces

This study has shed light on the fate of labeled aflatoxins in the gastrointestinal tract of animals and provided valuable insights into the excretion and distribution of aflatoxins in animals fed diets with and without yeast.

The results indicate that levels of radioactive molecules in the gastrointestinal tract decreased after 48 h of application, suggesting that biotransformation processes may have occurred, leading to the formation of intermediate and final products that were subsequently excreted from the organism, mainly through feces. These findings are consistent with previous research done by Ellis et al. (1991), Hsieh and Wogan (1982), and Swenson et al. (1974) that have demonstrated similar results.

The results indicate that fecal excretion of aflatoxins 24 h after application is consistent with previous findings by Wogan et al. (1967) who reported approximately 50% excretion in the first 24 h. In addition to the significant excretion of labeled aflatoxins through feces, our results showed that a portion of the radiomolecules was also eliminated through urine. This finding is consistent with previous studies by (Wogan et al. 1967; Baptista 2005) that have reported substantial excretion of aflatoxins through both feces and urine. Similarly, urinary excretion levels corroborate the results found by Baptista (2005) but differ from those reported by Wogan et al. (1967) who observed around 20% of total radioactivity excreted through urine in rats fed with AFB1^14^C-containing diets in the first 24 h. A more recent radioassay carried out by Firmin et al. (2010) showed that less than 6% was recovered in the urine and around 60% in the feces after 72 h. Possible reasons for this discrepancy could include variations in basal diet, animal conditioning temperature, or differences in the methodology of labeled toxin application.

The present study contributes to the existing knowledge about the metabolism and excretion of aflatoxins, as well as their effects on different organs of the body. Our findings are consistent with previous research conducted by several authors who demonstrated the absorption, metabolism, and excretion of labeled aflatoxins by the body (Wogan et al. 1967; Dalezios et al. 1973; Lüthy et al. 1980; Kumagai et al. 1998; Ellis et al. 2000; Baptista 2005). Our study also confirms that feces are the primary route of excretion of aflatoxins and their metabolites. Additionally, our investigation reveals a new and significant finding that the behavior of AFB1^3^H in animals fed with diets containing active dehydrated yeast Y904 is comparable to that of animals fed with a standard diet without the presence of this microorganism. This observation suggests that active dehydrated yeast Y904 may not influence the metabolism and excretion of aflatoxins in animals.

### Aflatoxin residence time in the intestines

Our study found that even at 72 h after the application of labeled aflatoxin B_1_, there was still detectable material from the toxin in the rats’ intestines. This suggests a slow clearance rate from this organ. Although the levels of labeled aflatoxins found in the intestine were low, it is important to note that these levels remained constant between 24 and 72 h in both treatments. These results raise concerns about the potential damage to intestinal cells during this period, especially given that previous researches has demonstrated the harmful effects of aflatoxins on various tissues (Heathcote and Hibbert 1978; Smith and Moss 1984; Baptista et al. 2002). It is possible that the slow clearance rate of aflatoxins from the intestine during this period could result in prolonged exposure of intestinal cells to these toxins, increasing the risk of damage and potential health consequences.

### Yeast control of aflatoxicosis

The results of this study demonstrate that the addition of active yeast to the diet naturally contaminated with aflatoxins significantly reduced the damage caused by these toxins to hepatocytes. This is consistent with previous studies by Baptista et al. (2001), Çelik et al. (2003), Parlat et al. (2001), and Stanley et al. (1993), who also found that active yeast has the potential to mitigate the harmful effects of aflatoxins in animals fed with contaminated diets. Additionally, Santin et al. (2003) reported that the use of yeast cell walls, obtained from breweries and physically separated, in the diet of broiler chickens resulted in a reduction of aflatoxin effects.

In this study, the histological analysis of the liver tissues from animals that received the aflatoxin-contaminated diet supplemented with active yeast showed a morphology similar to that of the livers of animals that received an aflatoxin-free diet, indicating that the yeast-added treatment effectively controlled the actions of aflatoxins in the cells. This finding is in agreement with the results reported by Baptista et al. (2004), Ellis et al. (1991), Heathcote and Hibbert (1978), Smith and Moss (1984), who observed significant hepatic damage in animals fed with diets contaminated with aflatoxins. These studies reported cell disorganization, the proliferation of biliary ducts, necrosis of the hepatic parenchyma, vacuolization, swollen hepatocytes, hydropic degeneration, high-fat levels, and pigment retention in the hepatic tissues of animals fed with aflatoxin-contaminated diets. Therefore, the addition of active yeast to the diet may be a promising approach for controlling the deleterious effects of aflatoxins on the liver.

### Potential mechanisms in damage control

In the literature, two hypotheses have been proposed to explain the aflatoxicosis control by yeasts. According to Karaman et al. (2005), the protective effects of yeast against aflatoxin-induced damage in broilers may be due to the presence of glucomannans in the yeast cell walls. Wu et al. (2009) and (Shetty and Jespersen 2006), citing Devegowda et al. (1996), stated that components of yeast cell walls called oligomannan after being esterified were able to bind 95% of the AFB1. Thus, the first hypothesis proposes that yeast sequesters the aflatoxins and promotes their excretion, preventing them from being absorbed by the gastrointestinal tract. However, most of the articles supporting this first hypothesis were assessing the binding ability of the glocomannan in in vitro experiments (Devegowda et al. 1996; Shetty and Jespersen 2006; Shetty et al. 2007; Hassan et al. 2021). In in vivo studies, Aravind et al. (2003) and Raju and Devegowda (2000, 2002) found improved performance and reduced aflatoxicosis effects in broilers subjected to a diet with aflatoxin and supplemented with esterified glucomannan. However, these last studies do not include radiomolecules nor assess the mitigation of the histopathological damages caused by aflatoxins. The results obtained in this study do not support the first hypothesis, as the distribution of aflatoxin B1 and/or its derivatives was the same in the presence and absence of active yeast, indicating that the yeast was not able to bind/sequester the aflatoxin and change its distribution in the rats’ bodies.

Baptista et al. (2004) carried out a bioassay with Wistar rats supplemented with inactive yeast (thermolysed) and manno-oligossacharides and found clear signs of toxicity and damage in the liver tissue, while the animals fed with dehydrated active yeast showed a lower intensity in the damages caused by aflatoxicosis in the liver. These findings are supported by authors that did not find positive results with the use of inactive yeast for the control of aflatoxicosis in in vivo studies (Baptista et al. 2002). Thus, yeast should be active to be used effectively as probiotic (Stanley et al. 1993; Parlat et al. 2001; Çelik et al. 2003).

Other studies suggest a second hypothesis that yeast may provide enzymes, vitamins, and unidentified growth factors that reduce the disturbances caused by the aflatoxins, possibly acting in the biotransformation of this mycotoxin in the organism (Crumplen et al. 1988; Krause et al. 1989; Baptista et al. 2001; Çelik et al. 2001, 2003; Parlat et al. 2001). These molecules can be amino acids, sulphates, or glutathione (Crumplen et al. 1988; Krause et al. 1989; Baptista et al. 2004), with glutathione being of particular interest due to its high levels in yeast (Alfafara et al. 1992a, b).

### The most plausible mechanism

Based on our results and in previous work in the literature we propose the following specific mechanism by which yeasts reduce the hepatic damages caused by aflatoxicosis.

The AFB1 acquire its mutagenic and carcinogenic characters after the epoxidation, in its AFB1 8–9 epoxide (epoxy form), as it can form covalent bonds with nucleic acids or Schiff bases with cellular and microsomal proteins, such as guanine, methionine, and histidine, being toxic and carcinogenic (Yiannikouris and Jouany 2002). However, the AFB1 8–9 epoxide can be conjugated with soluble nucleophilic molecules, as the enzyme S-glutathione-transferase (Yiannikouris and Jouany 2002), which effectively mediate the excretion and detoxification of the aflatoxin in its epoxy form (Paul et al. 2015). Thus, the presence of considerable amounts of S-glutathione-transferase could avoid the AFB1 8–9 epoxide to reach the liver and cause hepatic damages. The active yeast may provide this S-glutathione-transferase (Alfafara et al. 1992a, b; Jeppesen et al. 2003), reducing the toxicity effect of the aflatoxin in its epoxy form, as shown in Fig. 4.

### Practical applications

The worldwide aflatoxin contamination of food and animal feed is of a great concern (Jallow et al. 2021), with recent studies indicated the presence of mycotoxins, mainly aflatoxin, in detectable levels on 60 to 80% on global food crops (Eskola et al. 2020). Food and animal feed contaminated in levels above the established threshold may need to be used for other purposes, as fertilizers (Buenavista et al. 2021) and for biofuel production (Murthy et al. 2005). However, during corn ethanol production process with contaminated corn, only low amounts of aflatoxins are degraded and most of it remains in the distiller’s dried grains with soluble (DDGS), becoming a potential problem for animal feed (Lillehoj et al. 1979). Even during normal production processes on industrial scale, aflatoxins are found in the DDGS. Zhang and Caupert (2012) made a survey on 8 ethanol plants in the USA from 2009 to 2011 and detected aflatoxin in 12 of 64 samples (from 1 to 2 µg kg^− 1^). In Brazil the corn ethanol production is increasing considerable in recent years (Barros and Woody 2020) and a recent study detected aflatoxins in one third of DDGS samples from Brazilian plants (average 1.4 µg kg^− 1^). These values, however, are below the common accepted levels (Jallow et al. 2021).

In broiler production studies have already shown its effectiveness and economic viability (Afzal et al. 2022), as the yeast can be recovered from different industrial processes, as beer production (Bovo et al. 2015) and in the case of the corn ethanol industries, in the same plant contaminated DDGS can be produced together with yeast that can also be commercialized as probiotics for animal feed. Thus, the supplementation of *Saccharomyces cerevisiae* in the animal diets with potentially contaminated food should be recommended in order to reduce the risks for animal health. In addition to that, the use of active yeast as probiotic in human diet is also highly recommended (Sambrani et al. 2021; Abid et al. 2022).

## Conclusions

This study elucidates the excretion pattern and residence time of aflatoxins and/or its metabolites in Wistar rats, indicating that fecal excretion is the primary route for elimination of these toxins. The slow clearance rate of aflatoxins and its metabolites from the intestine raises concerns about potential damage to intestinal cells. The addition of active yeast to the diet contaminated with aflatoxins effectively controlled the actions of aflatoxins in the cells, as evidenced by liver tissue morphology similar to that of animals receiving an aflatoxin-free diet.

Therefore, this study adds to the existing knowledge about the metabolism and excretion of aflatoxins, and their effects on different organs, and confirms that active dehydrated yeast Y904 may not influence the distribution and excretion of aflatoxins in animals, indicating that the mechanism by which yeasts control aflatoxins is biochemical. Overall, the findings suggest that active yeast may be a potential therapeutic option as probiotic for mitigating the harmful effects of aflatoxins on liver cells.

## Data Availability

The data sets analyzed in the current study are available upon request from the corresponding author.
